# New Consumer Research Technology for Food Behaviour: Overview and Validity

**DOI:** 10.3390/foods11050767

**Published:** 2022-03-07

**Authors:** Garmt Dijksterhuis, René de Wijk, Marleen Onwezen

**Affiliations:** 1 Wageningen Food and Biobased Research, Wageningen University and Research, P.O. Box 17, 6700 AA Wageningen, The Netherlands; rene.dewijk@wur.nl; 2 Wageningen Economic Research, Wageningen University and Research, P.O. Box 9101, 6700 HB Wageningen, The Netherlands; marleen.onwezen@wur.nl

**Keywords:** consumer research technology, food choice, behaviour measurement, validity

## Abstract

Background: the last decade has witnessed an explosion of new consumer behaviour research technology, and new methods are published almost monthly. To what extent are these methods applicable in the specific area of food consumer science, and if they are, are they any good? Methods: in this paper, we attempt to give an overview of the developments in this area. We distinguish between (‘input’) methods needed to shape the measurement context a consumer is brought in, e.g., by means of ‘immersive’ methods, and (‘output’) methods that perform measurements proper. Concerning the latter, we distinguish between methods focusing on neuro-science, on psychology, and on behaviour. In addition, we suggest a way to assess the validity of the methods, based on psychological theory, concerning biases resulting from consumer awareness of a measurement situation. The methods are evaluated on three summarising validity criteria; conclusions: the conclusion is that behavioural measures generally appear more valid than psychological or neuro-scientific methods. The main conclusion is that validity of a method should never be taken for granted, and it should be always be assessed in the context of the research question.

## 1. Introduction

In recent, years many new technological research methods have been proposed, tested, and published that enable the study of consumer food behaviour. The developments follow each other very rapidly, and by now, a very diverse palette of research methods has become available for consumer researchers to choose from. On the one hand, this results in new and promising possibilities to study consumer food behaviour; on the other hand, it may also result in unclarity for researchers. It can be difficult to decide which methods are available for specific research questions and how useful and valid they are. An overview of a wide range of methodologies is needed. For food consumer scientists specifically, it is of interest to find out whether new methods are applicable in their area and if they provide valid measurement results.

A main question regarding new technologies is how to rate the validity of the new technologies. In particular, some of the novel technologies may change the way consumers are aware of being part of a study. This paper concerns the validity of new consumer research technologies, as applied in a food behaviour context. Therefore, we introduce three validity criteria based on psychological theory concerning biases resulting from the awareness a consumer has of a measurement situation (see Appendix A). The measurement methods introduced will be scored on these criteria in order to enable a view on the validity of the methods. First of all, the three criteria are introduced to spawn discussion concerning validity of consumer research. Such discussion on the validity of consumer research is needed (e.g., [1]), as was also pointed out by Dijksterhuis [2] in a critical review concerning the high failure rate of new food products.

### 1.1. Input and Output Technologies

The current study adds to the literature by providing an overview of novel technologies in the area of food consumer behaviour. We broadly differentiate between input and output technologies. Some technologies are used to bring the consumer into a certain situation in order to create a mind-set as close to real life as possible. Different contexts, operating at the input side of a study, are typically the independent variables of a study. Although we acknowledge the presence of different types of input, as noted, for example, by the recent so-called EPI-cube (Embodiment-Presence-Interactivity, [3]), we will not further differentiate between them.

Other new technologies are used to measure consumer related variables in order to get a grip on consumer behaviour or responses; these work at the output side of a study and typically are the dependent variables of a study.

Providing information to a consumer in an experiment (or to a respondent in a survey) can happen in many ways. The input side of consumer research consists of the experimental situation, which can include the screen of an online survey, the instruction by an experimenter, the physical surrounding during a measurement or any context provided by means of VR and related technologies.

At the output side of consumer behaviour, several types of new technologies can be used to measure behavioural outcomes. Here, we distinguish three types of methods aimed at collecting data on a neuroscience, a psychological, or a behavioural level.

Neuroscientific measurements, are measurements related to neural activity. They often refer to neurophysiological measurement, be they CNS (Central Nervous System) or ANS (Autonomic Nervous System) based. CNS-based measurements often employ such technologies as EEG, fMRI, or MEG, and they often point at some form of cognitive processing. Other psychophysiological measurements, i.e., of a wide range of ANS functions, often indicate the execution of tasks.

Psychological measurements inquire about psychological traits or states of a human subject—in our case, a ‘consumer’. Despite the seemingly broad range of methods that could fall under this heading, we define them as measurements of mental phenomena. Self-reported, past, or prospected behaviour is also considered a psychological measurement.

Behavioural measurements refer to the observable behaviour of a consumer. Any movement that is somehow monitored can constitute a ‘behavioural measurement’. Typically, these are motor measurements such as gait, movement, agitation, but hand movement, eye-movement, face movement, or chewing behaviour also fall under this heading. Response time measurements are also an example of a behavioural measurement.

One recent interesting innovation exists in voice assistants. Typically, one may think of voice assisted device operation (car navigation, phone number calling, (very) smart TV programming, [4]). One can also think of types of smart devices, such as Google Assistant or Siri. These devices can be used for consumer research by asking questions or giving instructions to consumers to operate their devices. At the same time, these smart devices may collect data in the form of the responses consumers give to their instructions or questions. Ethical problems obviously lurk, as it is not always clear if a consumer wants his/her voice commands and responses to be recorded for subsequent analysis, even if anonymously.

### 1.2. Implicit and Explicit Methodology

*Explicit* consumer research methods are those methods that require some form of answering from consumers, often using conscious reflection. Consumers have to make explicit what they mean, why they act as they do, why they make some choice, etc. Hereby, they are conscious of what they are answering, and they may ponder their answer before they give it. In contrast, *implicit* methods do not require consumers to do this. The implicitness, here, refers to the fact that information concerning consumer behaviour is inferred from a measurement without the consumer knowingly having control over the outcome (cf. [5,6]). As an example, the amount eaten and the speed with which food is consumed can be seen as implicit measures of acceptance of the food.

One may be tempted to think that implicit methods always require consumers being unaware of the measurement, but this is not true. It is possible to have consumers explicitly report on some matters but without the research question being addressed in this matter. The answers of the consumers can next be studied to provide evidence of their ideas, opinions, or attitudes concerning the matter. This type of measurement is implicit, although consumers are to explicitly answer some questions. In this case, the implicitness of a consumer measurement concerns the consumer being unaware of the underlying research question, rather than in the way consumers are to provide data (which appears explicit). In Section 4, such implicitness will be coupled with a method’s validity.

In De Houwer and Moors [7], it is argued that a measurement procedure (what we may call a research method in our context of food related consumer studies) cannot be named ‘implicit’. They rather reserve the term ‘implicit’ for measurement outcomes. This means that we could not talk about ‘implicit methods’ but of ‘implicit measurement outcomes’. De Houwer [8] suggested to equate ‘implicit’, in this sense, to the term ‘automatic’. However, most of the context of their discussions of implicitness are done in the context of (implicit) attitudes. This means that the constructs-to-be-measured are ‘implicit’ to the subjects. This applies to the IAT (Implicit Association Test, ref. [9] revealing implicit stereotypes, often used in the context of racial prejudices. In the context of our current paper, being food related consumer behaviour, the constructs-to-be-measured, themselves, are not necessarily to remain unknown to the subjects. Subjects may e.g., know they are consuming too much unhealthy food, to name an example. What they do not know is the underlying motivation for their food choice, so this motivation remains implicit. The type of research methods we refer to aim to understand, to explain, and, ultimately, to predict consumer food related behaviour.

Some authors seem to equate implicit measurements with physiological measurements. Often, such methods are indeed implicit, but implicit measurement is by no means restricted to this. De Wijk and Noldus [10], in their overview of implicit and explicit measures of emotions, list four (implicit) measurement types:

1. measures that reflect the activity of the central nervous system, such as EEG and fMRI,
2. measures of activity of the autonomic nervous system such as skin conductance and heart rate,
3. expressive measures, such as facial expressions,
4. behavioural measures.

One could argue that specific facial expressions, such as surprise, can also be the result of emotional processes and as such, partly fall under the second type, an autonomous reaction. The same can be said about behavioural measures. Posture or walking speed can be the result of, or correlate to, autonomic activity. What De Wijk and Noldus [10] make clear is that there seems to be no sharp distinction between implicit and explicit measurement.

In this paper, we suggest not to define implicit measurements in terms of the measurement process itself but rather, in terms of the way a measurement situation is set up.

## 2. Input Side: Context Providing Technologies

Food products are seldom consumed in isolation. Instead, foods are typically consumed in specific consumption situations, such as at home, in a restaurant, or at a canteen. These situations tend to shape our food experiences. Consequently, food preferences measured in a sensory laboratory may be different from preferences for the same foods measured in a real-life situation, as demonstrated by numerous studies. Many of these studies report higher preference ratings in real-world situations [11] or differences between several contexts [12,13], even though there are also some studies that report no difference [14].

Testing consumers in real-life consumption situations is often difficult because these situations tend to be noisy and offer little control over conditions, which may have unwanted effects on the measurements. Instead, there is a growing trend to recreate consumption contexts in the laboratory. Using so-called ‘immersive technologies’, these recreated contexts offer a compromise between real-life consumer experiences and tightly controlled laboratory conditions. ‘Immersion’ is a term used for what is thought to happen to consumers in a research situation where they are provided with a situational context that totally captures their attention—ideally to the point that they forget that they are in an experimental context. As advantages of ‘immersive technologies’, some have been mentioned:

1. overcoming the ‘respondent burden’ (the degree to which a consumer finds participation difficult, time consuming, or emotionally stressful [15],
2. increasing some statistical quality (validity, reliability, power) of the collected data [16],
3. adding control to an experimental situation, while keeping the situation ‘ecological’,
4. adding context to (online) surveys to enable more truthful (and hence valid) answers or to increase commitment from respondents.

In a way, telling a good story to a consumer, in an otherwise quiet testing room, can also be seen as an immersive ‘technology’. Reading a story from a paper or listening to a story told is so low-tech that we will not cover this in more detail here [17] (p. 30 on ‘Story Telling’ and p. 31 on ‘Sketchy descriptions’). In short, anything to have the subject mentally ‘leave’ the testing surrounding and enter the realm of their imagination can be seen as an ‘immersive’ technology. Short, specialised surveys have been developed to probe how deep consumers felt ‘immersed’ while being in such a situation [16].

In the below, we will introduce some recent (input) context providing technologies, ordered from a relatively low level of ‘reality’ (or ‘immersiveness’) to a high level.

### 2.1. Tabletop Technology: A Tablet Computer as an Expensive Coaster

**Figure 1:**
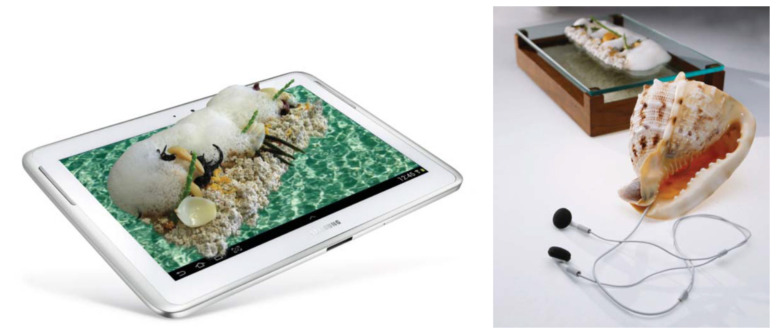
(**Left panel**): a tablet computer as a plate to hold your food. (**Right panel**): sound provision during eating (from [18], p. 316 and 329, resp.).

To provide consumers with a special eating context while dining, tablet computers have been used. They can provide a special context to the food, and it’s feasible to apply them in this manner in food testing (Figure 1, left panel). However, other ways may be possible that are less cumbersome or expensive. Other technology, e.g., sound, has been provided with food in a similar vein (Figure 1, right panel).

### 2.2. Virtualised Food Products

Using photogrammetry, a food product is totally visually digitalised and digitally recreated. The recreation may follow a (factorial) design, systematically varying aspects of the food product (e.g., its size, shape, colour, surface roughness, etc.), to be presented to consumers. The consumers next assess sensory properties, of course restricted to visual aspects, but they could assess expectations of other aspects, as texture or flavour. Gouton et al. [19] present an application using chocolate chip cookies and a comparison of simulated cookies with real cookies. Some differences were found, which Gouton et al. [19] suggest may depend on specific photogrammetric software settings.

### 2.3. VR Technology

**Figure 2:**
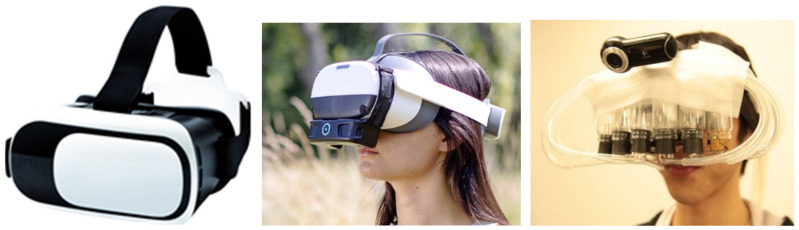
(**Left panel**): VR glasses, wherein one can slide a cell phone; (**Middle panel**) (reprinted with permission from OVR technology (Copyright OVR technology)) and (**Right panel**) (reprinted with permission from Takuji Narumi (Copyright Takuji Narumi)): a VR system enabling olfactory stimulation.

A plethora of VR-related technologies has seen the light in the last decade. VR-glasses have been around for a while, and they can now be obtained against relative low costs. Tools and devices exist where one can insert a cell phone in a device (Figure 2), and dedicated apps on the cell phone will assure a VR-presentation when wearing the device. Cardboard -fold your own- versions exist for under €1. These lend themselves for being sent to large groups of consumers, for in home, online survey testing, providing context through dedicated apps. Dynamic context, the typical VR-experience where one can virtually move around inside a surrounding, can be provided through these means. Many tools include integrated sound and, often, spatial sound effects.

Taufik, Kunz, and Onwezen [20] conclude that VR-provided contexts often lead to measurement results comparable to their real life counterparts. In addition, they conclude that the technology also seems promising to lead consumers to change their behaviour. Fang et al. [21] point out that VR methodology can also help reduce the hypothetical bias (the difference between a real experiment and an imagined one) by introducing a form of realism to the measurement situation.

Adding odour, relevant in a food context, to a virtual surrounding is more of a challenge. Several devices have been developed, but maybe some should be called contraptions instead (see Figure 2, rightmost panel). Advertisements exist, boasting about their ability to produce 1 ms short odour pulses and a time to switch between odours of 20 ms. When one realises that it may take some 300 ms, depending on many circumstances, for an odourant to reach olfactory receptors [22], such numbers look a bit over-the-top.

Other VR applications exist that use vibratory devices to simulate felt textures on the hand [23]. Straightforward applications in food related consumer science may not be in sight, or it must be the possibility to deliver vibratory stimulation in-mouth to simulate oral texture. Although technically feasible at this moment, it’s probably currently restricted to laboratory environments [24].

Bone conducting devices have been applied to record auditory and/or vibratory stimulation. In particular, in specific food related studies, where one may want to record the chewing sound as perceived by the chewer her/himself, and we know that chewing sound is, to a large extent, bone conducted sound [25,26]. They could also be used to provide food related auditory or vibratory stimulation, e.g., to adapt the sound perceived (vibrations felt) by consumers when chewing food (although we have not found papers in this area). A vibrating straw technology exists where no food is sucked through the straw, but a vibratory device can deliver the illusion of food streaming into the mouth [18].

**Figure 3:**
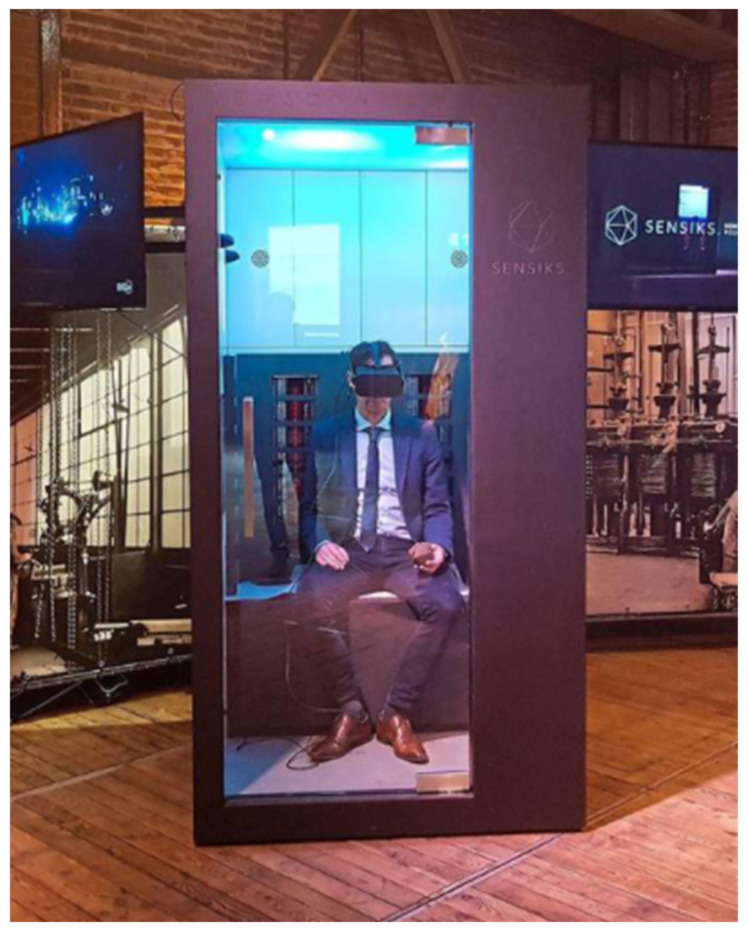
A subject sitting in a ‘Sensory Reality Pod’ (Reprinted with permission from SENSIKS. Copyright 2019 SENSIKS).

Another technology is the development of ‘Sensory Reality pods’ (Figure 3). Inside something that is best described as a ‘phone booth’, a subject takes place, puts on VR-goggles, and can, from within the Pod, be stimulated with sound, smells, air flows, and heat radiation. It provides a multisensory immersive surrounding. Applications in food science may be a bit farfetched at the moment, but they are certainly feasible. At this moment, one person at a time can be immersed, but in theory, several pods can be used simultaneously, if only cost were no issue.

### 2.4. AR Technologies

In AR (Augmented Reality) parts of the real physical surrounding of a consumer is integrated into the virtual surrounding. VR knows limited application in eating research or other applications due to the fact that consumers cannot comfortably interact with a real food. Many VR devices do not allow smelling, eating or drinking, and will spoil the immersion. In AR the interaction is provided through integrating an object into the virtual surrounding. A fully natural interaction with ones surrounding, in particular with the (food) object, is still not possible. The interaction will likely still feel alienating.

In the development of packaging, AR applications are clearly envisionable, as they allow visual interaction of a consumer with a packaging. Coupling visual AR to a haptic vibratory device (providing manual package texture simulation), one can imagine that the interaction may reach a level close to reality.

### 2.5. (Serious) Gaming Applications

All above mentioned examples can also be used in gamification applications. A computer game aims to give a subject a lively sense of immersion by providing a virtual context. Reality is not implied in the type of context (the game can be about the weirdest of worlds), but it is provided by the interaction with the environment by the moving and handling virtual objects, reactions of other persons (or alien entities) in the game, etc. The original aim of computer games is entertainment, but a shift to consumer research applications is feasible. Gamification has also been applied to make surveys more engaging [27]. This is also an application area that can be of use to any type of consumer research.

Applications in (food) marketing exist in an application where visitors of an entertainment park can plan ahead their visit, enabling the park to optimally locate their services [28]. In particular, in the context of online purchasing, there may appear a future for such applications.

Giving smell or taste feedback in gaming applications has also been suggested [29], although this appears a farfetched application, as, at this moment, it is still at a distance from use in consumer research.

### 2.6. Wall Projections/CAVEs

**Figure 4:**
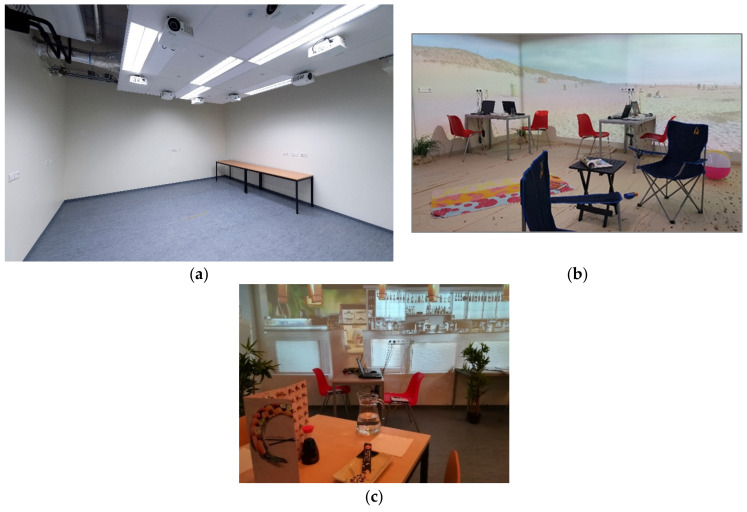
(**a**) WFBR ‘Experience room’. Lower: projections of a beach (**b**) and a sushi restaurant (**c**). Note that props are used in the room to add to the immersion. (Reprinted with permission from WFBR. Copyright 2019 WFBR).

The mentioned alienation in AR may result in it not yet being used much in food related applications or in typical consumer science applications where many consumers are tested. An alternative is the projection of a surrounding onto the walls of a room or in a ‘CAVE’. A CAVE (a recursive acronym: Cave Automatic Virtual Environment) is a (small) room, where, onto the walls, an environment is projected, either by back projection (where the walls need to be translucent), normal projections by means of beamers, or using very large LCD-screens. WFBR (Wageningen Food and Biobased Research, one of the research institutes of Wageningen Research, part of WUR.) employs a CAVE-like projection room, where eight beamers can project images onto the walls of a normal room (Figure 4a). In addition to the visual projections, sounds can be played, and an odour dispersing unit is installed as well.

Figure 4b shows the ‘Experience Room’ in action in a recent study where a beach environment was created. Note that props (towels, sand-coloured flooring) are used to add to the level of immersion in the simulated surrounding. The (c) panel of Figure 4 shows a recreated sushi restaurant, including real tables with menus and some (plastic) plants to help increase the reality level of the simulation [30,31].

One can imagine that there is no limit to the possibilities of providing (near real) projections with sound and odour. Room temperature, air humidity, etc., could be added, provided the budget to develop the technology is high enough.

### 2.7. ‘Non-Virtual’ Reality

A special case is the immersion in a rebuilt environment, as was done by Holthuysen et al. [11], who rebuilt an airplane fuselage in a lab room, to test air catered food items, complete with airplane engine noise. It was immersion, but it is not the type of consumer technology we’re addressing here.

## 3. Output Side: Measurement Technologies

In the sections below, several ways of collecting consumer related data are introduced, based on them collecting variables from neurophysiological, psychological, or behavioural origin. We will present different methods in the three areas mentioned. Some will be mentioned only scantily, as they may be very new, hardly used, or are on the fringe of what we can see as promising new consumer measurement methods in the food area.

### 3.1. Neuro Scientific Measurement Technologies

Neuroscientific variables can be of an overwhelming complexity, in addition to them being plentiful. They have in common that they attempt to measure neuronal correlates of consumer behaviour relevant to the area at hand. We will not introduce such techniques as EEG, fMRI, MEG, ANS-measurements in some detail, nor psychophysiology in general, but we list specific ways in which some of these technologies have recently been put to use to understand consumer food related behaviour. All these measurement techniques have that they require a connection to the human body in common. Although they do often not have to penetrate the skin or require otherwise invasive medical procedures, their impact on normal functioning mostly makes them listed as potentially invasive techniques. They will, thus, require some form of Ethical Clearance.

Innovation in food consumer neuro science may not only lie in developing new technology, in addition to the many existing methods that exist, but also in their combination. According to Niedziela and Ambroze [32], these methods should be used in addition to, not instead of, established food consumer methodology. It is useless to employ an expensive and complex measurement that is equivalent to liking, when a simple liking question may provide the same result.

Another innovation is taking neuroscientific measurements outside the lab into the real world, which has to do with making the technology portable. EEG-caps have become, more-or-less, portable and allow for this. Ambient EEG measures may introduce additional noise, possibly rendering measurement results even more difficult to interpret. In addition, the validity of EEG brain activity assessments is not always known. A recent paper shows that an unequivocal interpretation of EEG measures is not always possible. Eijlers et al. [33] measure arousal, resulting from looking at magazine advertisements (including food ads) using EEG, and conclude that their findings cannot be taken to show ad effectivity.

A recent neurophysiological development is fNIRS (functional Near InfraRed Spectroscopy) applied to brain activity. It is a technology where NIR radiation is sent through the skull by an optode and picked up after it has been reflected by brain tissue. The hemodynamic response of the brain tissue affects what can be picked up, which is related to the activity of the brain tissue. This technology has been applied in consumer research. It is claimed that it is more portable and easier to handle than other neurophysiological measurements. However, it appears to be, to date, removed from practical (portable) applications in consumer science, and it is a laboratory tool still (but see [34].

Augmenting online measurements with neuro scientific measurements (including ANS) has also become feasible. Using the camera in respondents’ (laptop) computers, heart beats can be inferred from a colour change of the face or forehead. Other ANS-devices, or even EEG, can be coupled to respondents’ computers, but this will bring some additional complexity still and is not yet applicable for large consumer samples.

**Figure 5:**
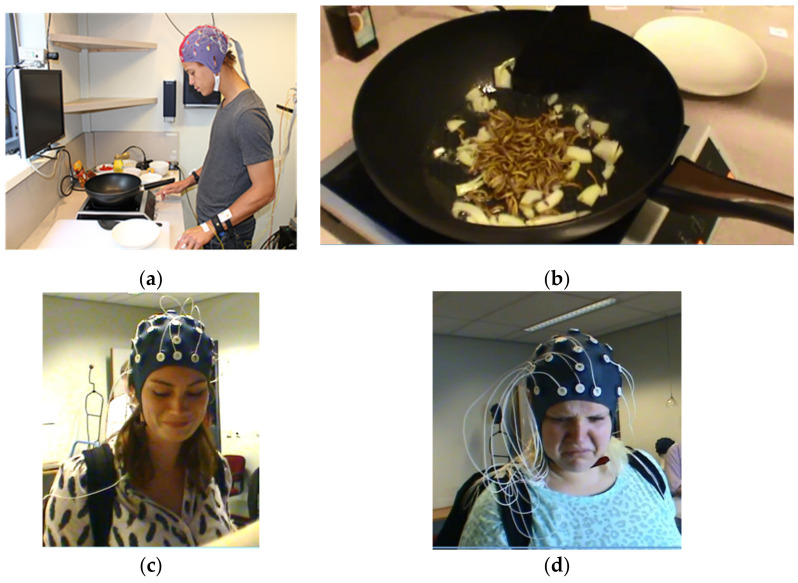
Subjects in the study of Brouwer et al. (2019): (**a**) subject cooking (photograph from [36], Figure 1, p. 5. Reprinted with permission from ref. [36]. Copyright 2019 A.-M. Brouwer); (**b**) dish with mealworms in the frying pan; (**c**,**d**) subjects’ faces on seeing the mealworms (Reprinted with permission from A.-M. Brouwer. Copyright 2019 A.-M. Brouwer).

An example of an innovative ‘field’ application combining several above mentioned (near) portable technology can be found in Brouwer et al. [35]. They combined measuring EDA (Electro Dermal Activity), ECG (Electro CardioGraphy) and EEG, allowing for extraction of several parameters, while their subjects were cooking and tasting. The subjects were voice-instructed to follow a strict cooking and tasting protocol. They cooked a meal with either chicken or mealworms, which was only revealed to them during cooking. Brouwer et al. [35] report they can, from the neurophysiological data, predict, with 82% accuracy, what dish a subject was cooking (mealworms or chicken). The aim of this study was to provide an implicit measurement method of affect or emotion during an actual cooking experience. In a follow up study, Brouwer et al. [36] collected measurements of facial expressions and wrist accelerometry in addition. Figure 5 shows some subjects in the Brouwer et al. study [36], obviously in a laboratory setting, not in their own kitchens.

#### Biological Measurement Technology

**Figure 6:**
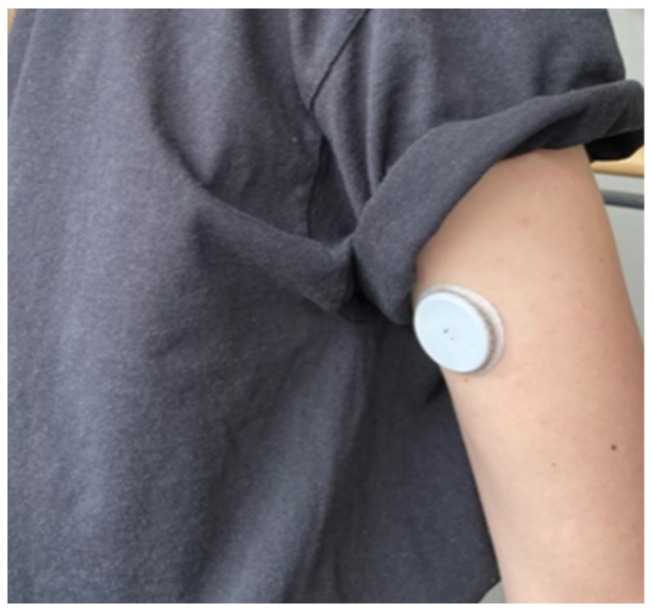
Wearable glucose sensor, to be worn on (and in) the arm. (Reprinted with permission. Copyright 2022 Marieke Ubachs).

For sake of completeness, we’ll list some recent developments that may not strictly fit the moniker of ‘neurophysiology’ but are biological in their origin. One recent development is a portable glucose level sensor (Figure 6). More of these types of sensors have become available, and they can link (via Bluetooth) to a smartphone app or to other devices. Although some say their device is ‘non-invasive’, a short needle is to penetrate the skin still. Nevertheless, consumers sometimes report that they forget they are wearing the device. Finding out if truly non-invasive versions [37] exist (and are reliable) will require additional literature search.

Some ‘smart watches’ can provide biological or psychophysiological data as well. Obviously, wearers of these devices are not continuously aware of the measurements being made.

Breathalysers have also been deployed to non-invasively measure physiological parameters from subjects. To what extent this is applicable, or has been applied, in a food related consumer context is not known to the authors. Obviously, it can potentially provide relevant diet-related information, including that related to flavour release, or flavour retention, in mouth or throat.

Another recent development, of quite a different nature, is what some may call ‘consumer genomics’, as in the paper by Masih and Verbeke [38], which covers the relationship between the expression of certain opioid receptors and the results of individuals on the PANAS mood scale [39]. To what extent claims in this field can already be translated to real life consumer behaviour in the food area remains to be studied.

### 3.2. Psychological Measurement Technologies

Any survey, or set of questions, can be given to a group of consumers, thus constituting a ‘psychological measurement’. Even when new questionnaires or new psychological scales are developed, the technology is hardly to be called new. Perhaps, with adaptive on line surveys, e.g., of the conjoint type, one can speak of some (technological) innovation. Newer developments are found in Big Data and AI technology that enable ‘very’ interactive surveying, where a path through a set of items may depend on the answers of many other respondents.

In this section, we will present several rather different technologies that aim to obtain information of consumers’ attitudes towards, or reported choices of, food. Many of the newer technologies are often online extensions of earlier developments. Sensometrics, covering statistics, data collection, and experimental design, is a rapidly evolving field from which we list a few innovations. AI and Big Data oriented applications form such a vast and rapidly expanding field that we decided not to include this area.

#### 3.2.1. Text and Web Scrape Technology

**Figure 7:**
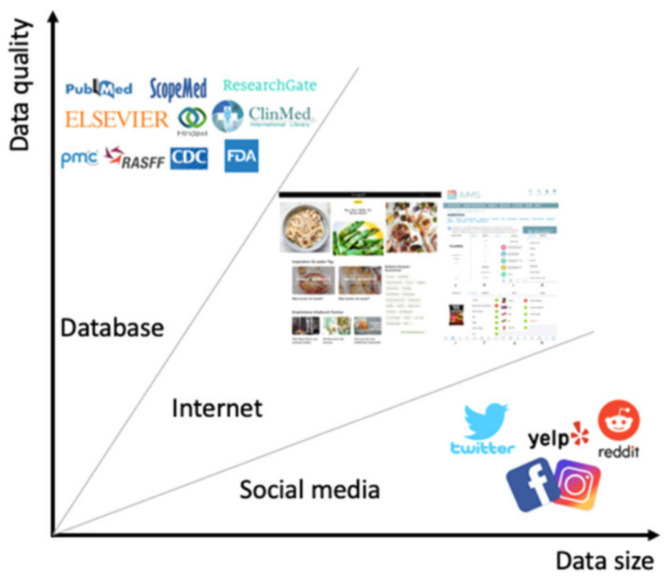
Text sources’ quality and size, for text mining purposes, in a food and nutrition context (Figure 2 from [41]).

Automatic interpretation of texts, either from the web or otherwise, made available is a relatively new and currently expanding technology, also called text mining, or text analytics. It is defined by Hearst [40] as ‘the discovery by computer of new, previously unknown information, by automatically extracting information from different written resources’. It has to be set apart from text search, as this refers to the finding of things you are looking for, so things that you already know something about (e.g., that they exist). Text mining aims to discover new things, previously unknown information, from any text source. It is already being applied in food consumer contexts [41]. In Figure 7 (taken from [41]), the size and quality of text sources is shown. It is presented here merely to provide an indication of the possibilities of the technology, as well as for consumer research in our field. The figure shows that the best quality is provided by the scientific publishers, and the lowest is by general social media sources. In between is the internet, where ‘anything’ can probably be found.

These days, text mining will likely also be possible from recorded spoken text, enabling even greater ‘mining’ possibilities.

Web-scraping is becoming a standard tool, provided by several agencies in many different contexts. A relatively recent application is the automated mining of food and recipe related data on the web. Another related technology concerns the analysis of texts from consumers that have been asked to describe a certain product. This text can be automatically processed [42]. In this way, automatic analysis of consumer prompted, written comments on specific products may reveal underlying ideas and feelings that consumers have about specific products.

#### 3.2.2. Surveys and Ecological Momentary Assessment (EMA)

Online survey research is not new. Every thinkable set of questions can be asked to consumers, both in offline and online surroundings. The easier it is to ask questions, the easier it is to answer them, and the more pregnant the following dictum becomes: “The biggest problem in asking consumers a question, is that you will get an answer” (after E.P. Köster, personal communication).

Additional measures of an implicit nature (mouse clicking, answering speed, etc.), can also be collected online, in addition to the answers given in the survey. These are behavioural/motor, rather than psychological, measurements.

Alerting consumers at certain moments to give an answer to a question, as is possible via smartphone technology, enables a new way of surveying. When consumers are required to list their activities or food consumption at random moments during the day, a bias that may arise from filling out questions at fixed moments on the day will be countered (e.g., using the Foodprofiler [43]) or the Traqq app, [44]). Another development is EMA (Ecological Momentary Assessment [45,46]), the sending of surveys to consumers’ smartphones when they are in a certain surrounding. Obviously, they have to first agree to their smartphone providing their location, and other parameters, to the investigators. If this is agreed, questions can be sent to consumers depending on a host of environmental parameters. Based on the location (in a supermarket, in a city, at a bus-station, etc.), time of day, weather conditions, previous activities (cycling, walking, shopping, relaxing), specific questions can be sent to the consumer. In this way, the investigators can ask questions appropriate to the real context the consumer is in. This technology also allows to interactively adapt a consumer’s set of questions, depending on the answers of another specific consumer or a consumer segment.

#### 3.2.3. Online Interaction with Consumers

Many ways exist to interact with consumers that are not in physical proximity, but they are online. Currently, there are online versions of many consumer face-to-face research methods.

In Table 1, traditional and online focus groups are compared (adapted from [47], Table 6.1, p. 137).

Traditional focus groups are a well-established tool for inventorying consumer opinions in a range of areas. Online focus groups [47] are a relatively new development. The participants in such a focus group attend the meeting from behind their computer, as does the moderator. Nowadays many people have, at least, some experience with online meetings, so online focus groups will not be alienating to most consumers. It must be added that video/audio connections only cannot replace the full gamut of non-verbal communication that a moderator will use in face-to-face meetings. Validity issues—not uncommon in focus groups—remain, and they will have to be closely scrutinised. Special software has been developed to aid the interpretation of focus group transcriptions [48].

Netnography is ethnographical methodology applied to consumers’ internet behaviour. The online activities of consumers can be surveyed, or otherwise investigated, all within the limits of privacy legislation, which can be a complicating and contentious area. In addition to individual consumers’ net-behaviour, consumers subscribing to communities and services can be studied. When, say, a consumer is following recipe-sites that contain vegetarian recipes, in addition to traditional ones, a food producer may be interested in knowing the reaction of this consumer community on certain plant based products to replace dairy.

#### 3.2.4. Sensometrical Methods

Sensometrics is the field that concerns itself with research methodology, statistics, and experimental design, especially aimed at collecting and analysing data in the sensory and consumer sciences (www.sensometric.org, accessed on 4 January 2022). The remit of the field reaches beyond food to include all instances where humans (in trained panels, or as consumers) are used to collect data concerning their perception, or liking, of product-related stimulation. Originally, these products were limited to food products and food-related stimuli, and the panels typically were sensory panels. Over the years, the field included ‘sensory and consumer’ studies of non-food products, including a wide a range of products, spanning home and personal care to cars. The Sensometric Society organises a conference every second year, and the contributions are published in the Elsevier journal Food Quality and Preference. They are a rich source of methodology and statistics in the area of sensory and consumer science. New methods, both for data collection and for data analysis, are continuously published, too much to even attempt to summarise in this review.

An example of a typical new(ish) method from this area is the technology whereby consumers can sort items on a screen. The position of the items after the sorting can be totally free, in groups, or following a pre-specified criterion (‘Sort these bottles into three groups based on which you find belong together.’). The numerical position on the screen or the group structure can be analysed using the apparatus of multivariate methods [49]. In particular, MDS [50] or other methods, allowing mapping and matching of spatial configurations of stimuli [51], are apt for the analysis of this type of data.

Almost every week, a new AI-based method is published to find structure in large data sets. Additionally, consumer (food) related data is collected in ever increasing amounts and with increasing speeds. AI-methods may be necessary to enable analysis of these amounts [52], there’s simply too much data for human analysts to process. This also applies to neuro scientific data. The data sets collected during fMRI-experiments are so huge and complex that automated pre-processing has become necessary. Data sets collected from the combination of neuro scientific, psychophysiological, and other consumer (food) behaviour measurement methods are of the same ilk: huge and complex. Automated, AI-based analysis methodology may be needed: powerful, but for many a practitioner, beyond their control. This control appears to be transferred from the content based psychologist or neuro scientist to the statistician/AI-specialist who designs and develops the software needed to analyse the data. It is, therefore, becoming ever so important that the validity of the measurement method and the forthcoming data can be guaranteed.

### 3.3. Behavioural Measurement Technologies

Any behavioural output can be seen as a (muscle) motor reaction to external stimulation. We list very different types of behavioural measurement technologies, of which we found a few food consumer related applications. They cover measurements very close to an individual (oral sensing, food ingestion, chewing behaviour), somewhat more remote (online) measurements (clicking behaviour, face reading, eye-tracking, reaction times, voice analysis), and automatic tracking of consumers when navigating an area.

#### 3.3.1. Food Ingestion and Automatic Eating Behaviour Assessment

Many innovations exist in the study of food consumption and food ingestion. This is the study of the amount, and types, of food that a consumer eats and digests. Food ingestion is notoriously hard to measure. See Willet [53] for the many different approaches in this area. When consumers report their own intake, it appears to be severely biased toward under reporting. Scientists in this field are always on the lookout for more objective, valid ways to measure actual intake. Many tools exist to measure ingestion, and they range from micro measurement instruments that may be inserted into a molar [54], to video registration of people eating, recording of muscle activity in the jaw, tongue, and/or throat, weighing plates while eating, or weighing the person after dinner.

A new development is the automatic recognition of what is on a plate, video capturing the plate, and submitting the images to software that is able to discern what is on the plate (and hopefully, indeed, will end up in the consumer). Software employing AI has been developed that can estimate the nutritional value of what is on the plate by processing the image [55].

The developers of the tooth mounted sensor [54] suggest it can intra-buccally monitor ingestion, to the point of sensing the nutritional quality of the material ingested.

Another innovation in this area is the extraction of additional, food consumption relevant parameters from the video capturing consumers’ faces. One of these parameters relates to the consumption duration, i.e., the time between putting the food in the mouth, and the last swallow. Other parameters that can be collected are chewing behaviour (duration of chewing, type (continuous or in bursts) of chewing, biting behaviour (estimated size of bite), etc. [56,57].

#### 3.3.2. Consumer Food Choice

Any situation in which a consumer chooses an alternative from among a set of items constitutes the output of a choice process. In this review, we limit ourselves to relatively recent developments in measurement methods concerning food choice. Most valid food related consumer choice concerns real food. Many choices between food pictures can be made, or between descriptions of food, but such a reported choice would, in our view, be a psychological, rather than a behavioural, measurement.

Collected data about mouse clicks and their timing may also contain valuable information about the choice process. Many online survey tools allow for these types of measurements. Creative developments in food choice and ingestion measurement have been published recently. One such development is the computerised manipulation of portion size, by adjusting the portion on a plate, as it appears on a computer screen [58,59].

#### 3.3.3. Face Reading

Automatic processing of facial expressions has become standard technology. Applications of this technology in food related consumer research are of a more recent nature. A consumer is seated in front of a camera, and often a computer screen. S/he can answer questions on the screen, look at pictures, or do other tasks, while the camera records the face of the consumer. Software is available to infer emotions from facial expressions. The emotion data can be used to study the reactions of consumers to the pictures or tasks provided on the screen.

A more recent innovation in this area is online face reading, where consumers are at home behind their own computers and perform the tasks, while the laptop camera is recording their faces [14]. Thomas et al. [60] summarised six points to take into account concerning automatic facial emotion reading. They refer to an earlier study by Mahieu et al. [61], where several items (perfumes, video advertisements, and chocolates) were studied using face reader technology. We have used the points put forward by Thomas et al. [60] to formulate five points of attention when performing online face reading studies:

1. results depend on the type of product and product category,
2. not all emotions show differences between products,
3. an individual baseline emotion measurement is advised and appears stable,
4. not all face reading software yields the same result,
5. the face should not be obscured (e.g., by glasses).

In particular, the fourth point is troublesome. If different software systems for automatic facial emotion reading do not agree, one cannot be sure what it is that is assessed by the software. This is a serious threat of the validity of automatic emotional measurement through facial expression. It should be mentioned that the techniques are continuously evolving with regard to required lighting, interference by glasses/beards, and interference by movements during talking and eating. As a result of this, the differences between various techniques will likely become smaller.

#### 3.3.4. Eye-Tracking

Eye-tracking is a well-established technology that is also in the consumer sciences. One of the assumptions often made, however, is that the object that is projected onto the fovea is also the object of greatest interest for the subject, as well as that this object has the greatest impact onto the behaviour (choice) of the subject. This assumption is not always granted, as parafoveal stimulation can also attract attention, and it can also affect perception outside awareness. Much research has been devoted to this in a reading context [62].

Eye-tracking can be carried out with a static subject, but the newest methodology enables eye-tracking, either from moving subjects [63] or from subjects in an immersive environment [64]. Developments in this area combine several measurements, such as eye-tracking and face reading [65].

#### 3.3.5. Reaction Times

The time a subject takes before reacting on a stimulus, making a choice, or answering a question is since long known to carry information about the cognitive processes (‘elementary mental organisation’ [66]) intervening between the registration of the stimulus and the result of a behavioural reaction to it. Reaction times (RT’s) can be recorded, with high precision, in experimental laboratory settings. They can also be provided with online surveys, or online choice tasks. More noise will be present in such online RT’s than when lab-recorded, but provided the *N* is large enough, the RT’s can be as valuable as the lab-collected ones. Precautions will have to be taken in keeping the noise at acceptable levels by deleting very long and extremely short RT’s. This is no different from laboratory collected RT data. See Woods et al. [67] for an overview of the issues with collecting RT data over the internet. Kochari [68] mentions that the online RT’s collected, in numerical cognition studies, were comparable to those from lab-based studies.

#### 3.3.6. Sentiment Analysis

Free text comments can be analysed to infer emotions of the provider of the comments [69]. More sources can be analysed for sentiment-content, using specially trained, machine learning, algorithms. An additional new field includes the automatic analysis of tone of voice, the speed of talking, and other speech properties. This allows for an emotion estimation based on voice utterings [70]. The authors could not find evidence that this is used in food consumer science, e.g., analysing the emotional connotation of consumers discussing food items or meals.

#### 3.3.7. Tracking and Recording of Movement

Technology exists to track peoples’ position and movement while they navigate through a space. In consumer science this has been used in retail or mall environments. In addition, movement speed and gait tracking is possible while navigating in a retail environment (real or simulated), trying to find ones groceries or other products [71].

Relatively new behaviours are the movements one makes with a finger on a touch screen, or a mouse while pointing at positions on a computer screen, when navigating web-pages, or during online (food) ordering. Timing, trajectories followed, velocity of mouse or finger movements, and even pressure exerted, may reveal a lot about the interaction between the information on the computer or smartphone screen and the consumer doing the navigation.

#### 3.3.8. Observation Technology

Observing consumers can be done in a host of circumstances, ranging from highly unnatural environments, where a consumer is in a lab to perform certain tasks, to very ‘ecological’, where consumers have no clue of being observed. The latter type of studies may run into ethical problems when consumers are filmed or photographed. When they are ‘just’ watched by observers, the impact onto their privacy may be limited. However, privacy legislation forbids the following of individuals to, e.g., find out what they choose in shops and how they may compare alternative products. New methodology exists where automatic tracking, more than just navigation through a shop or mall, is possible. The technology may even allow identification of subjects, by facial recognition and recognition of their gait, and monitor choice behaviour automatically.

## 4. Validity

New technologies clearly extend the possibilities to manipulate the testing environment and to measure consumer responses. With the increase in the amount of new measurement methods, another problem arises: viz., so the burden of the work shifts from the collection of the data to the analysis of the data and interpretation of the results. The dictum ‘Rubbish in, rubbish out’, still holds, so due attention to the measurement method and its validity is perhaps more needed than ever. We will follow the definition of validity as presented in Borsboom, Mellenbergh, and van Heerden [72], who state that a measurement of an attribute (an attribute being something in the real world that one desires to probe using a measurement method) is valid when the attribute exists in the real world and when variation in it causally affects variation in the measurement. The research problems in our area revolve around food choice, food consumption, sensory testing, consumer acceptance, etc. The question is when they can be expected to causally affect our measurements. In order to establish this, a theory is needed that is able to couple the attribute to the measurement outcome [72].

We will approach validity in its most simple guise, viz. ‘A measurement is valid when it measures what you intend it to measure’, i.e., when it clearly relates to the matter under study. We claim that our field aims to find research methods to address our main research questions, viz. to understand food related behaviour and perception, and we aim to address these in a valid manner. This means that, when a method is employed to ‘measure’ consumer behaviour, it should clearly relate to that behaviour and not just to what consumers self-report their behaviour to be. It may also mean that a brain imaging study may not be able to predict ultimate revealed preferences of consumers. We know that there are many external threats to valid measurements. In particular, as consumers never operate as automatons in an information vacuum, we know that there are many biases and unwanted influences affecting their responses. This is the reason that the three criteria that we reiterate below, introduced earlier by Dijksterhuis [73], explicitly address consumers in a measurement situation. These validity criteria distinguish between levels of awareness as being part of a measure. We argue that, especially these levels of awareness, may change due to the application of novel research tools, as they result in novel ways to engage with consumer. We add to the literature by applying these validity criteria on a range of novel technologies, thereby providing researchers with guidelines to support their choices on whether and how to use these technologies.

### Three Criteria for Validity

The three criteria are not intended to disqualify any research method. They do not specify their strict application; rather, they are intended as a rule of thumb, enabling researchers to get an idea of the validity of a host of different methods. They may prompt them to find a method that best fits their specific behavioural research question. The criteria measure to what level of detail a consumer is aware of the measurement situation he or she is in. The idea being that such awareness may interfere with the result of the measurement. The psychological basis for this idea is briefly introduced in Appendix A.

The three criteria addressing validity are:

1. Reflection: the research method requires the ‘person(a)’ of the consumer, i.e., he/she needs to think about his-/herself or his/her behaviour,
2. Awareness: the method requires the consumer to know he or she is being tested,
3. Informed: the method requires the consumer to know the underlying research question.

In Table 2, we have listed several of the newer consumer science measurement (‘output’) technologies introduced in the above. For each of the presented research methods, a criterion applies or does not apply. This is shown in Table 2, where a method is given a tick mark (✓) in the appropriate column when the criterion applies.

Obviously, the scoring of the criteria is not an exact matter. It can be discussed, and it will depend on the exact way measurements are performed and research situations are constructed. This is also one of the main points in the whole exercise: that it should be discussed and that the validity of consumer measurements should never be taken for granted. The framework can, therefore, also be regarded as a guiding tool to reflect on and support decision making.

Some methods (criterion 1, ‘reflection’) require subjects to reflect on their own situation and past, future, or even hypothetical behaviour. This is the case in many survey or interview oriented methods. This is also underlying the well-known difference between stated and revealed preference in economics. In revealed preference theory, the measurement does not interfere with the consumer, as it only considers what is actually been bought or chosen.

In some methods, it is inescapable that the consumer knows he/she is being tested (criterion 2, ‘awareness’), e.g., it is hardly possible to measure psychophysiological parameters without the consumer knowing that a measurement is performed. Knowing to be in a test can influence the way consumers behave. However, the research question itself need not be disclosed in these measurements.

Regarding criterion 3 (‘informed’), there are types of methods, e.g., group discussions, in which consumers are directly interviewed about their view on the research question, so they have to know even the research question, or they can’t be part of the discussion.

Looking at Table 2, a number of things may be concluded. Obviously, it is impossible to perform neuro scientific tests with consumers without them knowing that they are in a test situation. The only exception may be the possibility to assess heart rate from a visual image of the face. Other ANS measurements require an apparatus that is impossible to apply surreptitiously. However, recent developments enable sensors, such as smart watches, that need to be worn for longer periods but appear to not bother the subject, and they often even forget they are wearing a sensor.

Psychological methods are so ubiquitous that it is impossible to even attempt to summarise many of them. We limited ourselves to some of the newer methods. Finding novel information in available texts by means of ‘web-scraping’, typically texts available on the internet, does not need any consideration of the producers of the text. Netnography may also be employed without consumers knowing they are being tested. Surveys, focus groups, and other methods where questions are asked do require consumers to have at least some information about the question they are inquired about.

All behaviour based methods presented here do not require a consumer to think about him- or herself to enable a measurement of their responses, nor do they need to know the research question. For example, the way one eats (food ingestion, eating behaviour), or what one chooses to eat (food choice), can be assessed by video without the eating consumer knowing. Tracking consumers’ movement, e.g., through a retail environment, also does not necessarily require this. Face reading, eye-tracking, reaction time measurement, or choice outcomes do not require a consumer knowing about the measurement taking place, although with eye-tracking, it is probably difficult to perform measurements unobtrusively.

Overall, the tick marks in Table 2 seem to hint at the conclusion that behavioural measurements appear more valid than neuro scientific measurements and psychological measurements. We hasten to say that these numbers are based on our particular choice of consumer measurement technology and on our interpretation of the technology. The finding does not reflect any superiority of one method over another. Other authors may use other definitions of their specific research methodology, and they may be confronted with research questions that demand specific applications of methodology not taken into account in our scoring.

## 5. Conclusions

With the above mentioned provisos, we tentatively conclude that the behavioural based methods, in general, appear to enable valid results, concerning actual consumer behaviour. When behavioural research data are collected in order to predict future consumer behaviour, behaviour based data may be the preferred type to base predictive models on. Psychological research methods can back such models up with behavioural knowledge and knowledge about consumer segments, based on psychological traits. The neuro-science based research methods are probably best suited to study very specific research questions in a small group of consumers.

All in all, the validity of (food) consumer measurement methodology should never be taken for granted. The three criteria provide a means to suggest the validity of a method and, perhaps more importantly, they show that validity should be discussed and taken into account before the measurements proper take place.

## Figures and Tables

**Table 1:** Comparison of traditional focus groups and their online version (adapted from [47], Table 6.1, p. 137).

	Traditional Focus Groups	Online Focus Groups
Interactivity among participants	✓	✓
Real-time client viewing	✓	✓
High-quality viewing experience	✓	
Full view of body language/facial expressions	✓	
High-quality audio-video recording	✓	
Video accessibility (e.g., archiving, clipping, replay)	✓	
Reduced travel for clients		✓
Reduced travel for moderators		✓
Regional diversity of participants		✓
Communication through computer, tablet, or smartphone		✓
Longitudinal perspective allowed		
Opinion and insights shared anywhere		
Multiple segments can be represented simultaneously and compared		
Flexible timing		

**Table 2:** Selected ‘new’ consumer science technologies and an indication of their validity, based on the three criteria. When a criterion applies, a tick mark (✓) is shown.

Measurement Technology	Criterion Applies		
	Reflection	Awareness	Informed
**Neuro/bio-methodology**			
neuro science			
EEG		✓	
fMRI (MEG)		✓	
fNIRS		✓	
ANS measurement			
GSR		✓	
heart rate		✓	
wearable glucose sensor		✓	
smart watch		✓	
consumer genomics	✓		
**Psychological**			
adaptive surveys	✓	✓	
EMA		✓	
(free) sorting	✓	✓	
consumer text analysis	✓		
text/web scraping			
online focus group	✓	✓	✓
netnography			
voice assistants (a la Siri)	✓	✓	✓
context in online surveys	✓	✓	
**Behaviour**			
screen/mouse movement			
food ingestion/eating behaviour		✓	
plate sensing			
buccal sensors		✓	
video eating behaviour		✓	
food choice			
portion/plate adjustment	✓	✓	
emotion measurement			
facial emotion reading		✓	
movement			
tracking navigation			
speed, gait, agitation			
eye-tracking		✓	
observation (automatised)			
reaction time		✓	
tone of voice			

## Appendix A. A Theoretical Basis for the Biases Resulting from Consumer Awareness of a Measurement Situation

In the chapter called “The methods and snares of psychology.” William James writes about ‘the named state’ which should be distinguished from ‘the naming state’ [74]. This means that a verbal report of a certain psychological state, say a felt sadness (the ‘named state’), is made while the subject is in another state (the ‘naming state’). A subject has to interpret his/her bodily and/or psychological circumstances and internal state to be able to, after the fact, report something such as ‘I am sad’, or ‘I currently feel sadness’. The reporting is a certain psychological state in itself, which will exert an influence, e.g., via a memory, on the previous state which one tries to report. James’ conclusion is that the introspection and report of one’s own internal psychological states is a snare.

It has become more and more clear that behaviour is not always the result of consciously willing it. Wilson [75] writes about the ‘adaptive unconscious’, shaped by evolution, guiding us through a complicated environment, and helping us with decisions through intuition. This all happens without our explicit knowledge, and often even without the possibility of such knowledge. Many of the psychological processes responsible are inaccessible to conscious awareness. In this context Wilson [75,76] states “People can no more observe how they are unconsciously categorizing their environments, setting goals, and generating intuitions than they can observe how their kidneys work.”

Damasio [77] explains the difference between an emotion and a feeling, much as James [74] has introduced this distinction. The former is a bodily reaction to a stimulus, and the latter a conscious interpretation of that emotion by the subject. This means that the emotion is attributed a reason, but this does not mean that this attribution is the very cause of the emotion; reasons are not causes. The underlying, unknown, causes may provide a non-rational route to decision making, alongside the (often fabricated) reasons resulting from a rational route. Kahneman and Tversky [78,79] have amply illustrated that in many economical decision making contexts most people make decisions that can be shown to be sub-optimal from a strict rational, utilitarian, viewpoint. As a result subjects may actually gain less money in comparison with the alternative they do not choose. Damasio [80] suggests that this is the result of an ‘emotional route’ to decision making which may supersede the rational route.

Another line of work showing the importance of the unconscious in perception and appreciation is that of Zajonc [81]. Lazarus [82] argues that it is necessary first to analyse a situation, and to build some knowledge about it, in order for an affective response to occur, an approach known under the name of appraisal theory. Zajonc [83] argues the opposite, emotion/affect is primary, only afterwards may knowledge about a situation occur, but cognition is not a prerequisite for affective responses to occur. A respectable number of studies show that emotion has primacy over cognition [84,85,86]. In one telling experiment subjects were tachistoscopically presented with words, flashed too short to enable identification. However, this short presentation still allowed an appropriate affective response to the words. The affective value of the stimulus is somehow registered but the identity of the stimulus remained unknown to the subjects [81].

## References

[1] Palascha A., van Kleef E., de Vet E., van Trijp H.C. (2021). Self-reported sensitivity to physiological signals of satiation and hunger: Assessment of construct validity. Pers. Individ. Differ..

[2] Dijksterhuis G. (2016). New product failure: Five potential sources discussed. Trends Food Sci. Technol..

[3] Flavián C., Ibáñez-Sánchez S., Orús C. (2019). The impact of virtual, augmented and mixed reality technologies on the customer experience. J. Bus. Res..

[4] Perez S. (2019). Retrieved from Tech Crunch. Techcrunch.com/2019/02/12/report-voice-assistants-in-use-to-triple-to-8-billion-by-2023/.

[5] Köster E.P. (2003). The psychology of food choice: Some often encountered fallacies. Food Qual. Prefer..

[6] Köster E. (2009). Diversity in the determinants of food choice: A psychological perspective. Food Qual. Prefer..

[7] De Houwer J., Moors A., Wittenbrink B., Schwarz N. (2007). How to Define and Examine the Implicitness Of Implicit Measures. Implicit Measures of Attitudes.

[8] De Houwer J., Wiers R.W., Stacy A.W. (2006). What are Implicit Measures and Why are We Using Them?. The Handbook of Implicit Cognition and Addiction.

[9] Greenwald A.G., McGhee D.E., Schwartz J.L.K. (1998). Measuring Individual Differences in Implicit Cognition: The Implicit Association Test. J. Personal. Soc. Psychol..

[10] De Wijk R.A., Noldus L.P.J.J. (2021). Using implicit rather than explicit measures of emotions. Food Qual. Prefer..

[11] Holthuysen N.T., Vrijhof M.N., de Wijk R.A., Kremer S. (2017). “Welcome on board”: Overall liking and just-about-right ratings of airplane meals in three different consumption contexts-laboratory, re-created airplane, and actual airplane: HOLTHUYSEN et al. J. Sens. Stud..

[12] A Willems A., van Hout D.H., Zijlstra N., Zandstra E.H. (2013). Effects of salt labelling and repeated in-home consumption on long-term liking of reduced-salt soups. Public Heal. Nutr..

[13] Meiselman H., Johnson J., Reeve W., Crouch J. (2000). Demonstrations of the influence of the eating environment on food acceptance. Appetite.

[14] De Wijk R., Kaneko D., Dijksterhuis G., van Zoggel M., Schiona I., Visalli M., Zandstra E. (2019). Food perception and emotion measured over time in-lab and in-home. Food Qual. Prefer..

[15] Bradburn N. (1978). Respondent Burden. Proceedings of the Survey Research Methods Section of the American Statistical Association.

[16] Hathaway D., Simons C.T. (2017). The impact of multiple immersion levels on data quality and panelist engagement for the evaluation of cookies under a preparation-based scenario. Food Qual. Prefer..

[17] Köster E., Mojet J. (2018). Complexity of Consumer Perception.

[18] Spence C., Piqueras-Fiszman B. (2014). The Perfect Meal: The Multisensory Science of Food and Dining.

[19] Gouton M.-A., Dacremont C., Trystram G., Blumenthal D. (2020). Validation of food visual attribute perception in virtual reality. Food Qual. Prefer..

[20] Taufik D., Kunz M.C., Onwezen M.C. (2021). Changing consumer behaviour in virtual reality: A systematic literature review. Comput. Hum. Behav. Rep..

[21] Fang D., Nayga R.M., West G.H., Bazzani C., Yang W., Lok B.C., Levy C.E., Snell H.A. (2020). On the Use of Virtual Reality in Mitigating Hypothetical Bias in Choice Experiments. Am. J. Agric. Econ..

[22] De Wijk R.A. (1989). Temporal Factors in Human Olfactory Perception. Ph.D. Thesis.

[23] Bueno M.-A., Lemaire-Semail B., Amberg M., Giraud F. (2014). A simulation from a tactile device to render the touch of textile fabrics: A preliminary study on velvet. Text. Res. J..

[24] Iwata H., Yano H., Uemura T., Moriya T. Food simulator. Proceedings of the ICAT 2003.

[25] Dacremont C. (1995). Spectral composition of eating sounds generated by crispy, crunchy and crackly foods. J. Texture Stud..

[26] Dacremont C., Colas B., Sauvageot F. (1991). Contribution of air-and bone-conduction to the creation of sounds perceived during sensory evaluation of foods. J. Texture Stud..

[27] Puleston J., Sleep D. (2011). The Game Experiments: Researching How Gaming Techniques Can Be Used to Improve the Quality of Feedback from Online Research.

[28] Lemon K.N., Verhoef P.C. (2016). Understanding Customer Experience Throughout the Customer Journey. J. Mark..

[29] Obrist M. Lecture at EuroSense 2020. https://esn-network.com/news/single-view/article/eurosense-2020-summary/.

[30] Van Bergen G., Zandstra E., Kaneko D., Dijksterhuis G., de Wijk R. (2021). Sushi at the beach: Effects of congruent and incongruent immersive contexts on food evaluations. Food Qual. Prefer..

[31] Zandstra E., Kaneko D., Dijksterhuis G., Vennik E., De Wijk R. (2020). Implementing immersive technologies in consumer testing: Liking and Just-About-Right ratings in a laboratory, immersive simulated café and real café. Food Qual. Prefer..

[32] Niedziela M.M., Ambroze K. (2020). The future of consumer neuroscience in food research. Food Qual. Prefer..

[33] Eijlers E., Boksem M.A.S., Smidts A. (2020). Measuring Neural Arousal for Advertisements and Its Relationship With Advertising Success. Front. Neurosci..

[34] Duncan K.K., Tokuda T., Sato C., Tagai K., Dan I. (2019). Willingness-to-Pay-Associated Right Prefrontal Activation During a Single, Real Use of Cosmetics as Revealed by Functional Near-Infrared Spectroscopy. Front. Hum. Neurosci..

[35] Brouwer A.-M., Hogervorst M., Grootjen M., van Erp J., Zandstra E. (2017). Neurophysiological responses during cooking food associated with different emotions. Food Qual. Prefer..

[36] Brouwer A.-M., Hogervorst M.A., van Erp J.B., Grootjen M., van Dam E., Zandstra E.H. (2019). Measuring cooking experience implicitly and explicitly: Physiology, facial expression and subjective ratings. Food Qual. Prefer..

[37] Hanna J., Bteich M., Tawk Y., Ramadan A.H., Dia B., Asadallah F.A., Eid A., Kanj R., Costantine J., Eid A.A. (2020). Noninvasive, wearable, and tunable electromagnetic multisensing system for continuous glucose monitoring, mimicking vasculature anatomy. Sci. Adv..

[38] Masih J., Verbeke W. (2019). Exploring Association of Opioid Receptor Genes Polymorphism with Positive and Negative Moods using Positive and Negative Affective States Scale (PANAS). Clin. Exp. Psychol..

[39] Watson D., Clark L.A., Tellegen A. (1988). Development and validation of brief measures of positive and negative affect: The PANAS scales. J. Pers. Soc. Psychol..

[40] Hearst M. (2003). What Is Text Mining?. https://people.ischool.berkeley.edu/~hearst/text-mining.html.

[41] Tao D., Yang P., Feng H. (2020). Utilization of text mining as a big data analysis tool for food science and nutrition. Compr. Rev. Food Sci. Food Saf..

[42] Symoneaux L., Cayla C., Anneraud P., Chretien G., Masson F., Lourtioux C., Coulon-Leroy N., Pouzalgues R. Automatic textual analysis of wine sensory characteristics based on tasters description. Proceedings of the Eurosense 2020 Symposium.

[43] Van de Puttelaar J., Onwezen M. (2016). Inzicht in Consumentenkeuze Voor Sierteelt: Kennisintegratiedocument PPS Consument, Keuzearchitectuur en Communicatie voor Sierteeltproducten. https://www.wur.nl/en/Research-Results/Research-Institutes/Economic-Research/Research-topics-WEcR/Consumer-Food/FoodProfiler-provides-insight-into-who-eats-food-and-what-where-why-and-how-it-is-eaten/FoodProfiler-Researcher.htm.

[44] Lucassen D.A., Brouwer-Brolsma E.M., van de Wiel A.M., Siebelink E., Feskens E.J.M. (2021). Iterative Development of an Innovative Smartphone-Based Dietary Assessment Tool: Traqq. J. Vis. Exp..

[45] Shiffman S., Stone A.A., Hufford M.R. (2008). Ecological momentary assessment. Annu. Rev. Clin. Psychol..

[46] Maugeri A., Barchitta M. (2019). A Systematic Review of Ecological Momentary Assessment of Diet: Implications and Perspectives for Nutritional Epidemiology. Nutrients.

[47] Burns A.C., Veeck A., Bush R.F. (2020). Marketing Research.

[48] Guerrero L., Xicola J., Ares G., Varela P. (2018). New Approaches to Focus Groups. Methods in Consumer Research.

[49] Courcoux P., Qannari E., Faye P. (2015). Free Sorting as a Sensory Profiling Technique for Product Development.

[50] Borg I., Groenen P. (2005). Modern Multidimensional Scaling: Theory and Applications.

[51] Gower J.C., Dijksterhuis G.B. (2004). Procrustes Problems.

[52] Martens H., Wulvik A., Fuglerud S.S. Quantitative Intuition: Combining prior knowledge and big data. Proceedings of the Sensometrics Conference 2020.

[53] Willett W., Willett W.C. (2013). Food frequency methods. Nutritional Epidemiology.

[54] Tseng P., Napier B., Garbarini L., Kaplan D.L., Omenetto F.G. (2018). Functional, RF-Trilayer Sensors for Tooth-Mounted, Wireless Monitoring of the Oral Cavity and Food Consumption. Adv. Mater..

[55] Weesepoel Y., Alewijn Daniels F., Baart M., Müller-Maatsch J., Simsek-Senel G., Rijgersberg H., Top J., Feskens E., Beyerer J., Längle T. (2021). Towards the universal assessment of dietary intake using spectral imaging solutions. Proceedings of the OCM 2021-Optical Characterization of Materials: Conference Proceedings.

[56] A de Wijk R., Engelen L., Prinz J.F. (2003). The role of intra-oral manipulation in the perception of sensory attributes. Appetite.

[57] de Wijk R.A., Janssen A.M., Prinz J.F. (2011). Oral movements and the perception of semi-solid foods. Physiol. Behav..

[58] Forde C.G., Almiron-Roig E., Brunstrom J.M. (2015). Expected Satiety: Application to Weight Management and Understanding Energy Selection in Humans. Curr. Obes. Rep..

[59] Forde C.G., McCrickerd K.K., Cheon B. (2015). Singa-Portion: Understanding Energy Selection and Intake in Asia Poster. Soc. Study Ingestive Behav..

[60] Thomas A., Brient M., Mahieu B., Teillet E. Facial expression measurement as a standard consumer test? Several technical points. Proceedings of the Eurosense 2020 Symposium.

[61] Mahieu B., Visalli M., Schlich P., Thomas A. (2019). Eating chocolate, smelling perfume or watching video advertisement: Does it make any difference on emotional states measured at home using facial expressions?. Food Qual. Prefer..

[62] Schotter E.R., Angele B., Rayner K. (2011). Parafoveal processing in reading. Atten. Percept. Psychophys..

[63] Hummel G., Maier S., Baumgarten M., Eder C., Strubich P.T., Stroebele-Benschop N. (2021). Visual attention towards food during unplanned purchases—A pilot study using mobile eye tracking technology. PLoS ONE.

[64] Siegrist M., Ung C.-Y., Zank M., Marinello M., Kunz A., Hartmann C., Menozzi M. (2019). Consumers’ food selection behaviors in three-dimensional (3D) virtual reality. Food Res. Int..

[65] Gunaratne N.M., Fuentes S., Gunaratne T.M., Torrico D.D., Ashman H., Francis C., Viejo C.G., Dunshea F.R. (2019). Consumer Acceptability, Eye Fixation, and Physiological Responses: A Study of Novel and Familiar Chocolate Packaging Designs Using Eye-Tracking Devices. Foods.

[66] Luce R.D. (1984). Response Times: Their Role in Inferring Elementary Mental Organisation.

[67] Woods A.T., Velasco C., Levitan C., Wan X., Spence C. (2015). Conducting perception research over the internet: A tutorial review. PeerJ.

[68] Kochari A.R. (2019). Conducting Web-Based Experiments for Numerical Cognition Research. J. Cogn..

[69] Visali M., Mahieu B., Thomas A., Schlich P. (2020). Automated sentiment analysis of Free-Comment: An indirect liking measurement?. Food Qual. Prefer..

[70] Dasgupta P.B. (2017). Nirma Institute of Technology Detection and Analysis of Human Emotions through Voice and Speech Pattern Processing. Int. J. Comput. Trends Technol..

[71] De Wijk R.A., Maaskant A.M., Kremer S., Holthuysen N.T.E., Stijnen D.A.J.M. (2018). Supermarket shopper movements versus sales and the effects of scent, light, and sound. Food Qual. Prefer..

[72] Borsboom D., Mellenbergh G.J., van Heerden J. (2004). The Concept of Validity. Psychol. Rev..

[73] Dijksterhuis G.B. Diversity in Methods in Sensory Consumer Science: Key-Note at ‘Sense of Diversity’. Proceedings of the Second European Conference on Sensory and Consumer Science of Food and Beverages.

[74] James W. (1890). The Principles of Psychology.

[75] Wilson T. (2001). Strangers to Ourselves: Discovering the Adaptive Unconscious.

[76] Dijksterhuis D. (2012). The total product experience and the position of the sensory and consumer sciences: More than meets the tongue. New Food Mag..

[77] Damasio A.R. (2011). Fundamental Feelings. Nature.

[78] Kahneman D., Tversky A. (1979). Prospect Theory: An Analysis of Decision under Risk. Handbook of the Fundamentals of Financial Decision Making: Part I.

[79] Kahneman D., Tversky A. (2020). Choices, Values and Frames.

[80] Damasio A.R. (2003). Looking for Spinoza: Joy, Sorrow and the Feeling Brain.

[81] Zajonc R. (1980). Feeling and Thinking: Preferences need no inferences. Am. Psychol..

[82] Lazarus R.S. (1984). On the Primacy of Cognition. Am. Psychol..

[83] Zajonc R.B. (1984). On the Primacy of Affect. Am. Psychol..

[84] Bechara A., Damasio H., Tranel D., Damasio A.R. (1997). Deciding Advantageously Before Knowing the Advantageous Strategy. Science.

[85] Bechara A., Damasio H., Damasio A.R. (2000). Emotion, Decision Making and the Orbitofrontal Cortex. Cereb. Cortex.

[86] Kihlstrom J.F., Pervin L.R., John O. (1999). The Psychological Unconscious. Handbook of Personality.

